# Adoption by olive baboons (*Papio anubis*) of newly constructed electricity pylons as sleeping sites in Laikipia, Kenya

**DOI:** 10.1002/ece3.11164

**Published:** 2024-03-12

**Authors:** Laiyon Lenguya, Lolimo Ewaton, Nicholas W. Pilfold

**Affiliations:** ^1^ Loisaba Conservancy Nanyuki Kenya; ^2^ Sanctuary Tambarare Ol Pejeta Conservancy Nanyuki Kenya; ^3^ Conservation Science & Wildlife Health San Diego Zoo Wildlife Alliance San Diego California USA

**Keywords:** crop raiding, electrocution, olive baboon, parasite exposure, predation avoidance, pylons, sleeping sites, thermoregulation

## Abstract

Olive baboons (*Papio anubis*) use fixed, secure, and naturally occurring sleeping sites such as tall trees and rocky cliffs, as protection from predators and often show a selection preference for particular trees or rocky cliff faces. We documented olive baboons' adoption of recently constructed high‐tension electrical transmission towers (pylons) as a novel type of sleeping site in Laikipia, Kenya. The use of pylons suggests that the greatest potential benefits may include reduced parasite exposure and predation avoidance. Thermoregulation and feeding efficiency are not supported as benefits because pylons increase baboons' exposure to wind and cool nighttime temperatures and the pylons were constructed in locations independent of established feeding sites. These observations advance our understanding of olive baboon sleeping site selection in a changing landscape.

## Graphical abstract

In Laikipia, Kenya, olive baboons (*Papio anubis*) were observed utilizing 45‐m‐tall high‐tension electrical towers (pylons) as sleeping sites. This new behavior enhances our understanding of baboons' natural choice of sleeping sites, which typically include trees and cliffs.

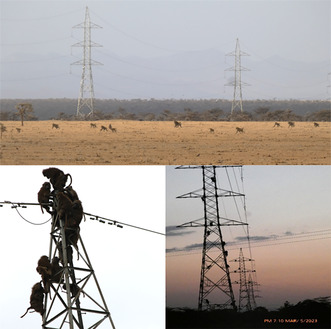

## 1 INTRODUCTION

Olive baboons (*Papio anubis*, hereafter “baboon”) use secure and naturally occurring sleeping sites such as tall trees and cliffs as an antipredator strategy while sleeping (Hamilton, 1982; Isbell et al., 2018). However, they often show a preference for particular trees or rocky cliff faces, by sleeping more often at some sites than at others (Bidner et al., 2018; Markham et al., 2016). In addition to predation risk, thermoregulatory benefits (Anderson & McGrew, 1984; Koops et al., 2012), parasite infestation and fecal‐matter load within sleeping sites can influence their frequency of use (Hausfater & Meade, 1982), and the limited availability of good quality sites is generally thought to regulate baboon distribution and forage access (Cowlishaw, 1997; Hamilton, 1982; Markham et al., 2016; Suire et al., 2020). While other primate species have been observed using anthropogenic structures such as rooftops and electricity poles (Bracken et al., 2021; Brotcorne et al., 2014; Hoffman & O'Riain, 2012), this behavior has not been reported before in Laikipia, Kenya (Bidner et al., 2018; Butynski & De Jong, 2014; Danish & Palombit, 2014; Isbell et al., 2017; Matsumoto‐Oda, 2015; Strum, 2005; Suire et al., 2020). For example, at Mpala Research Centre in Laikipia, despite the availability of pylons from the same transmission line within known baboon home ranges, baboons still use cliffs and tall trees as their preferred sleeping sites (Bidner et al., 2018; Isbell et al., 2018; Suire et al., 2020) and have yet to be observed using pylons. We document olive baboons using high‐tension electrical transmission towers (hereafter “pylons”) recently installed to transmit power between Kenya and Ethiopia (EEPCO & KETRACO, 2012) as sleeping sites, and we discuss the implications of this behavior for understanding natural sleeping site preferences.

## 2 MATERIALS AND METHODS

Observations were collected on March 5–6, 2023, at the 255 km^2^ Agriculture Development Corporation (ADC) Mutara ranch within Laikipia County, Kenya (Figure 2). Laikipia County is a 9700 km^2^ region of semi‐arid bushland in central Kenya, with a mean annual temperature of 18.3°C (range: 13.0–25.2°C), and a mean total of 812 mm of precipitation annually (monthly mean range: 23–133 mm) (Fick & Hijmans, 2017). ADC Mutara is in the southern part of the greater Laikipia‐Samburu ecosystem, which is a mosaic of grassland, *Euclea* shrubland, as well as *Acacia* and riverine woodland (Woodroffe & Frank, 2005). The property is served by two semi‐permanent rivers, the Suguroi River and the Mutara River, both with riverine habitat. Tall *A*. *xanthophloea* densely occurs along the Suguroi River (Nash & Whitten, 1989), lying between Mutara River and the pylons' used as sleeping sites. Approximately 3 km from those pylons', tall *A*. *xanthophloea* sparsely occurring along the seasonal Segera River have typical baboon sleeping site characteristics (Bidner et al., 2018; Isbell et al., 2021) and baboons have been observed utilizing them as sleeping sites (LL pers. Obs.).

The newly erected pylons intersect Mutara, Suguroi, and Segera Rivers in an east–west direction. The project, dubbed the Ethiopia–Kenya Transmission Interconnection Line, was commissioned in 2006 and launched in 2016 to export up to 2000 MW of electricity (EEPCO & KETRACO, 2012; Tadesse, 2022). The electric pylons are 45 m high, with a square base pillar spread of 17 m and cross arms extending to 15 m (EEPCO & KETRACO, 2012). These arms provide areas where baboons can rest and sleep.

On the evening of March 5 between 18:00 and 19:00, we opportunistically encountered baboons roosting on pylons during ongoing large mammal surveys by vehicle. After encountering them, we scanned the troops, counting, and aging individuals. We used a Garmin eTrex 10 Global Positioning System (GPS) to record the locations of the roosting sites. We used Empire Model 218 Binoculars (7 × 35) to enhance counting individual baboons in each troop, and a Canon EOS 1300D camera to take photographs to document observations. We returned to the sleeping sites on the morning of March 6th at 06:30, to confirm presence as an overnight sleeping site, and estimate baboon group sizes. We classified troops as separate groups based on distinctly different movement directions the following day. Independent baboon groups are known sometimes to share sleeping sites (Danish & Palombit, 2014).

## 3 RESULTS

**FIGURE 1:**
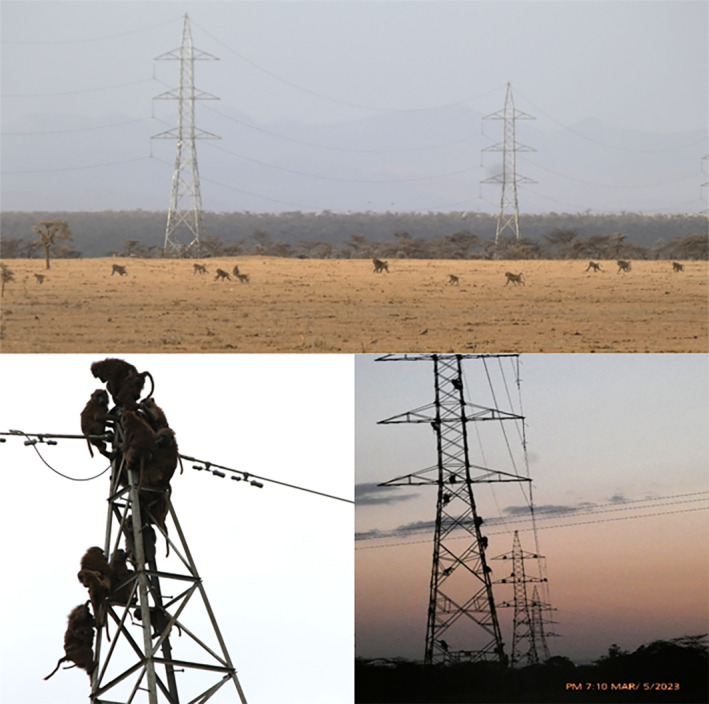
Three troops of olive baboons (*Papio anubis*) were observed on March 5, 2023, using recently installed high‐tension electric pylons as sleeping sites within *Acacia* woodland‐savannah in Laikipia County, Kenya.

**FIGURE 2:**
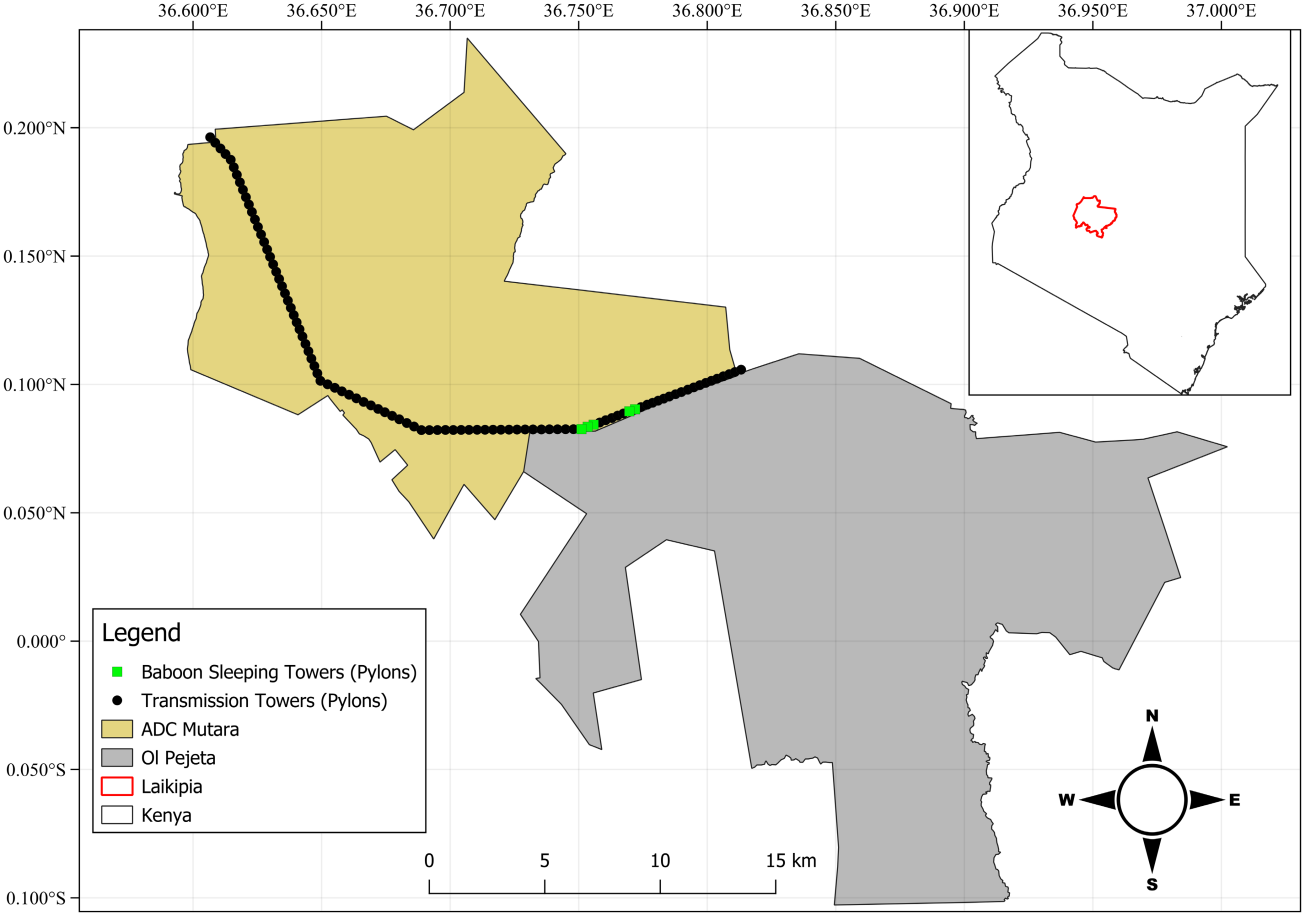
Map of ADC Mutara Ranch and adjacent Ol Pejeta Conservancy showing the High‐Tension Electrical Transmission Towers (Pylons) and their use by olive baboons (*Papio anubis*) as sleeping sites in Laikipia, Kenya.

We recorded three troops using pylons as sleeping sites in two distinct locations. The first sleeping site consisted of three different pylons and two troops (N. 0.08278°, E. 36.75079°) see (Figure 1; Table 1). We encountered another baboon troop using pylons as a sleeping site 2.5 km away (N. 0.0909°, E. 36.77193°). We observed two pylons occupied by baboons, but visibility was minimal given low light, and we could not identify demographics at each pylon. All baboons were still atop all pylons at 06:30 on March 6 when we arrived, confirming that all three baboon troops used the pylons as sleeping sites. Total troop sizes were estimated that morning: group one 80 individuals, group two 60, and group three 50 (Table 1). At the first sleeping site, the first group crossed to Ol Pejeta Conservancy, while the second group foraged inside ADC moving northward (Figure 2). While at the second sleeping site, baboons were observed foraging along the pylons inside ADC Mutara.

**TABLE 1:** Observed baboon groups demographics and the use of high‐tension electric pylon as sleeping sites in March 2023 at the ADC Mutara Ranch in Laikipia, Kenya.

Date	Time	Baboon troops	Used pylons	Group size	Demographics				
					Adult male	Adult female	Sub‐adult	Juvenile	Infant
05/03/2023	18:00	Troop 1	Two	26	4	8	5	7	2
05/03/2023	18:15	Troop 1		28	3	10	3	8	4
05/03/2023	18:25	Troop 2	One	45	5	12	10	14	4
06/03/2023	19:00	Troop 3	Two	No light	N/A	N/A	N/A	N/A	N/A
06/03/2023	6:50	Troop 1	Two	~80	Estimate of group size atop pylons				
06/03/2023	7:05	Troop 2	One	~60	Estimate of group size atop pylons and ground				
06/03/2023	7:20	Troop 3	Two	~50	Estimate of group size resting on the ground				

## 4 DISCUSSION

We report a rare observation of three troops of olive baboons utilizing electrical transmission pylons as sleeping sites in Laikipia County, Kenya. Usually, in this landscape, tall *Acacia* trees and rocky cliffs are predominantly selected as sleeping sites by baboons (Bidner et al., 2018; Hamilton, 1982; Suire et al., 2020). Typically, tall *A*. *xanthophloea* with vines and branches overhanging rivers remain preferred sleeping site (Bidner et al., 2018; Isbell et al., 2021). The ADC Mutara landscape is flat and mostly covered by short *Vachellia drepanolobium* (Figure 1); however, dense patches of tall *A*. *xanthophloea* occur along the Suguroi River (Nash & Whitten, 1989), and sparsely at both Mutara and Segera Rivers where baboons were observed previously using as sleeping sites (LL pers. Obs.).

Our observations, while limited, offer insights into sleeping site choices made by olive baboons. Normally, baboons sleep in tall trees or on rocky cliff faces. It has been suggested that these sites are chosen to (1) minimize thermoregulatory costs (Ellison et al., 2019; Koops et al., 2012); (2) be closer to foraging areas (Cowlishaw, 1997; Schreier & Grove, 2013); (3) minimize exposure to gastrointestinal parasites from feces (Hausfater & Meade, 1982); and (4) minimize predation, mainly from leopards (*Panthera pardus*) (Bidner et al., 2018; Hamilton, 1982).

Thermoregulatory benefits do not appear to explain the use of pylons, since the pylons are engineered to minimize wind drag (Jeddi et al., 2023) and, therefore, are less likely than trees and cliffs to shield baboons at night from wind‐induced heat loss. Foraging efficiency does not appear to explain sleeping site preferences either, since the pylons were constructed without regard to foraging areas and the baboons still slept in them. A study of baboon ranging behavior before and after pylons were constructed might shed more light on this. Baboons may be selecting pylons to minimize parasite and predator exposure. The pylons' limited surface area may allow feces to drop to the ground more readily, minimizing exposure to gastrointestinal parasites. Moreover, the very tall height and metal design of the pylons are likely to make them difficult for leopards to climb. Pylons also lack the branching structure and foliage cover of trees that can create ambush opportunities for leopards. If pylons indeed afford baboons greater protection from parasites or predators, we predict that baboons elsewhere along the transmission line will also adopt pylons as sleeping sites.

Baboons are known to crop raid (Strum, 2010). With the pylons traversing natural habitat such as ADC Mutara ranch and extending into small‐scale farms, they may encourage baboon crop‐raiding behavior, potentially resulting in heightened human–wildlife conflicts in this region. Electrocutions from use of anthropogenic power infrastructure have been documented in five species of primates in Kenya including yellow baboons (Katsis et al., 2018), but the survival risk posed by high‐tension electrical pylons as sleeping sites remains unknown. Future research is encouraged to investigate the potential costs of the introduction of the transmission line to humans (via increased crop raiding) and baboons (via electrocution) and to determine if improved safety from predators outweighs the risk of electrocution.

## AUTHOR CONTRIBUTIONS

**Laiyon Lenguya:** Conceptualization (lead); data curation (lead); investigation (lead); methodology (lead); writing – original draft (lead). **Lolimo Ewaton:** Conceptualization (lead); data curation (lead); investigation (lead); methodology (lead); writing – original draft (supporting). **Nicholas W. Pilfold:** Conceptualization (lead); data curation (lead); investigation (lead); methodology (lead); writing – original draft (supporting).

## CONFLICT OF INTEREST STATEMENT

The authors declare no conflict of interest in the publication of this manuscript.

## Data Availability

All raw data are available in Table 1.
